# Immunohistochemical study of dental pulp cells with 3D collagen type I gel in demineralized dentin tubules *in vivo*

**DOI:** 10.17305/bjbms.2020.4614

**Published:** 2020-11

**Authors:** Sultan Zeb Khan, Sana Mirza, Samina Karim, Takashi Inoue, Mohammed S. Bin-Shuwaish, Laila Al Deeb, Khold Al Ahdal, Rana S. Al-Hamdan, Ahmed M. Maawadh, Fahim Vohra, Tariq Abduljabbar

**Affiliations:** 1 Department of Clinical Pathophysiology, Graduate School of Tokyo Dental College, Tokyo, Japan; 2 Department of Oral Pathology, Faculty of Dentistry, Ziauddin University, Karachi, Pakistan; 3 Department of Ophthalmology, Hayatabad Medical Complex, Khyber Girls Medical College, Peshawar, Pakistan; 4 Department of Restorative Dentistry, College of Dentistry, King Saud University, Riyadh, Saudi Arabia; 5 Department of Prosthetic Dental Sciences, College of Dentistry, King Saud University, Riyadh, Saudi Arabia

**Keywords:** Collagen type I gel, demineralized dentin tubules, dental pulp cells, osteogenesis

## Abstract

Dental pulp cells (DPCs) represent good candidates for the regeneration of dental tissue. This study aimed to evaluate the growth and differentiation potential of DPCs cultured inside demineralized dentin tubules *in vivo*. Six green fluorescent protein-transgenic rats (body weight 100 g each) and thirty-two Sprague-Dawley (SD) male rats (body weight 250 g each) were used for DPC collection and dentin tubules preparation and transplantation, respectively. Third-passage DPCs with or without collagen gels were loaded into demineralized dentin tubules. Both types of grafts were transplanted into the rectus abdominis muscles of SD rats and were harvested after 21 days. The expression of alkaline phosphatase (ALP), bone sialoprotein (BSP), osteopontin (OPN), nestin, and dentin sialoprotein (DSP) was analyzed by immunohistochemistry. Histological analysis showed that DPCs in the collagen gel formed an osteodentin-like hard tissue matrix after 21 days. Increased positive immunoreactivity for ALP, BSP, OPN, nestin, and DSP was observed in experimental groups compared with control. Our results demonstrate that DPCs in collagen gel inside demineralized dentin tubules show increased growth and differentiation.

## INTRODUCTION

Caries is a common problem in dentistry, which is characterized by denatured dentin with consequent exposure of the dental pulp. The goal of operative dentistry and endodontics is the regeneration and repair of damaged dentin; and dental pulp plays a critical role in maintaining the homeostasis of teeth and the subsequent quality of life. Dental tissue that is damaged by disease or injury must be replaced, and traditional therapies using calcium hydroxide as a dental pulp capping agent are effective in inducing a reparative dentinogenic response [1]. In addition, microleakage at the tooth restoration interface can allow bacteria to infiltrate the dental pulp and cause restorations to fail. A natural barrier is provided by reparative/regenerative dentin for dental pulp tissue produced by stimulation from artificial restorative material [2]. Differentiation of the odontoblast-like cells and formation of reparative dentin is observed in experimental or clinical resection of the dental pulp along with the application of several dental materials in capping or pulpotomy procedures [3].

Pulp tissues within teeth are highly complex mesenchymal tissues containing odontoblastic cells surrounded by hard-mineralized tissue. Dental pulp mesenchymal stem cells are used in regenerative cell therapy as a component of endodontic treatment for carious lesions [4]. Previous studies have demonstrated that dental pulp stem cells (DPSCs) can differentiate into odontoblast-like cells to generate dentin-like mineral structures, both in *in vivo* and *in vitro* environment [5].

Scaffolds are commonly used to provide a 3-dimensional (3D) substrate for developing cells and a template for tissue engineering [6]. Polymers have shown good potential as scaffold materials because of their design, flexibility, and degradability. Synthetic polymers and poly-L-lactic acid (PLLA), in particular, are frequently used in tissue engineering. Synthetic polymers are incapable of cellular signal recognition; however, natural polymers such as gelatin are capable of inducing signaling and differentiation [7]. In an earlier study, Khan et al. cultured third-passage dental pulp cells (DPCs) in collagen gels embedded within mineralized dentin tubules, encouraging their development into osteoblasts [8].

Dentin is formed by a special set of cells known as ­odontoblasts, which reside along the dentino-pulpal junction. During tooth development, these cells are differentiated from neural crest-derived mesenchymal cells which are regulated by sequential interaction between neural-crest-derived mesenchymal cells and epithelial cells in the oral environment [9]. The fibroblast growth factor (FGF) family, which includes transforming growth factor-β (TGF-β) and insulin-like growth factor-1 (IGF-1), mediates interactions between epithelial and mesenchymal tissue that regulate proliferation, differentiation, and development in the early stages of tooth development. Similarly, bone morphogenetic protein (BMP) family members play an essential role in organogenesis, dentinogenesis, odontoblast differentiation, and tooth pattern formation [9]. In addition, it is reported that dentin matrix also contains a sequence of growth factors [10]. Therefore, demineralized hard tissues including dentin matrices and bone have osteoinductive characteristics when implanted in bone marrow, muscle, or collagen tissue. Furthermore, studies have indicated the dentinoinductive effects of demineralized dentin matrix on dental pulp sites [11].

Therefore, in the present study, the aim was to evaluate the hard tissue formation inside demineralized dentin tubules after implantation into the rectus abdominis muscles of rats using histology and immunohistochemistry. The characteristics of the mineralized tissue and the differentiation process of formative cells were determined. The origin of hard tissue formative cells was also analyzed by transplantation of green fluorescent protein (GFP) in transgenic rat DPCs.

## MATERIALS AND METHODS

### Ethical statement

This study was performed according to the guidelines set for the treatment of experimental animals at the Tokyo Dental College (Ethical Review Committee # 223207).

### Animals and tissue preparation

Six GFP-transgenic rats (body weight 100 g each) for DPCs collection and thirty-two Sprague-Dawley (SD) male rats (body weight 250 g each) for dentin tubules preparation and transplantation were used. All animals were sacrificed by cervical dislocation. The maxillary and mandibular molars of the GFP rats were extracted with forceps under induced anesthesia by intraperitoneal injection of sodium thiopental (0.3 ml/100 g). Cell culture and transplantation were performed as described by Khan et al. [8] in their mineralized dentin tubule study. Briefly, extracted molars were washed and cleaned with wash medium, which consisted of α- minimum essential medium (α-MEM; GIBCO, Paisley, UK) containing 50 μg/ml gentamicin (Sigma-Aldrich, MO, USA) and 0.3 μg/ml Fungizone (GIBCO). Attached periodontal ligament and enamel matrix were removed from each molar mechanically using a knife. After primary culture, the GFP rat DPCs were subcultured for up to three passages.

### Dentin tubules preparation

Dentin tubules were prepared from the mandibular and maxillary incisors of thirty male SD rats, each weighing 450 g. The apical end of each extracted incisor was cut with a band saw, leaving the incisal end closed. Pulp tissue was completely removed from the tubule using a barbed broach (06) (Mani, Inc., Utsunomiya, Japan). After removal of the pulp tissue, the incisors were first washed thoroughly with distilled water, then with 30% H_2_O_2_ (Wako, Osaka, Japan) and finally with saturated sodium hypochlorite solution (Wako, Osaka, Japan). This process was repeated 3 times. The tubules were then washed with a large amount of distilled water in a beaker using a magnetic stirrer at 4°C for 12 hours followed by defatting with 70% ethanol for 30 minutes and then with ether for 15 minutes. Dentin tubules were demineralized in 10% ethylenediaminetetraacetic acid (EDTA) solution to express BMP on the surface of each dentin tubule. The dentin tubules were then washed with distilled water, followed by 70% ethanol and then with ether (10 minutes). The length of each dentinal tubule ranged from 8 to 10 mm and width from 2 to 3 mm. These tubules were used as a container for the transplantation of DPCs with or without collagen gels.

### Preparation of grafts

Trypsin/EDTA solution was used to separate thrice-passaged DPCs. The separated DPCs were then centrifuged and suspended in α-MEM (supplemented with 50 μg/ml gentamicin, 0.3 μg/ml Fungizone, 10% FBS). Two types of graft materials were made for transplantation: 1) DPCs suspended in α-MEM and 2) DPCs in collagen type-1 gel (at approximately 5 × 10^4^ cells). The pre-prepared demineralized dentin tubules were then filled with the two types of the above-mentioned transplantation graft materials. Both types of grafts were prepared inside dentin tubules as described below.

1. Experimental: demineralized dentin tubules + GFP DPCs + collagen type 1 gel.
2. Control: demineralized dentin tubules + GFP DPCs + α-MEM (supplemented with 5g/ml gentamicin, 0.3 μg/ml Fungizone, 10% FBS).

Observations were carried out for each group at 21 days. Implantation of the experimental and control grafts was performed according to Khan et al. [8].

### Histological and immunohistochemical analysis

Rats were sacrificed at 21 days after implantation of dentin tubules. The implanted tubules along with the contiguous soft tissue were excised and fixed in neutral buffered formalin solution for 1 week. Fixed samples were washed with distilled water and later desiccated in different percentages of alcohol solutions and embedded in paraffin. After embedding, the samples were sectioned sagittally to a thickness of 4 μm. After fixation, decalcification, and embedding in paraffin, the sections were used for hematoxylin and eosin (H and E) and immunohistochemical staining.

To inactivate endogenous peroxidase activity, sections were treated with 30% H_2_O_2_ and methanol in a solution of phosphate-buffered saline (PBS) at room temperature. Blocking of the samples was done with 1% bovine serum albumin (BSA) in PBS for half an hour. Incubation of the samples was performed with alkaline phosphatase [ALP] (dilution 1:200 concentration), bone sialoprotein [BSP] (dilution 1:100 concentration), osteopontin [OPN] (dilution 1:1500 concentration), DSP (dilution 1:200 concentration), and nestin (dilution 1:100 concentration) primary antibodies in PBS containing 1% BSA for 1 hour and then later washed. Samples were incubated with biotinylated secondary antibody, Nichirei-Histofine simple-stain MAX-PO (Nichirei, Tokyo, Japan), for 45 minutes. After washing the samples 3 times with PBS, they were stained with Nichirei-Histofine simple-stain 3,3’-diaminobenzidine [DAB] (Nichirei) and were counter-stained with hematoxylin. Paraffin sections were observed using an Upright Zeiss Axiophot microscope (NY, USA).

## RESULTS

### Localization of GFP-positive cells

**FIGURE 1:**
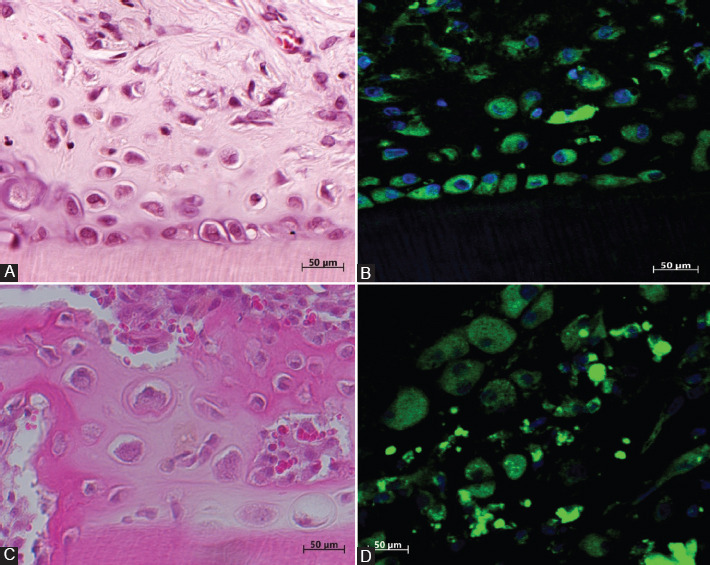
(A) Hematoxylin and eosin (H and E) staining of osteodentin-like hard tissue formed by dental pulp cells (DPCs) in the collagen gel after 21 days in the experimental group; (B) newly formed osteodentin inside demineralized dentin tubules. Osteoblast/odontoblast-like cells are showing positive immunofluorescence staining for green fluorescent protein in the experimental group; (C) H and E staining in the control group. DPCs formed a bone matrix-like hard tissue inside the walls of demineralized dentin tubules. H and E staining in the control group; (D) positive immunofluorescence staining of the osteoblasts inside the bone newly formed by DPCs without a collagen gel in the dentin tubules from the control group.

GFP rat DPCs were detected in both the experimental and control groups at 21 days by confocal laser microscopy and fluorescence staining. Three weeks later, all DPCs indicated fluorescence staining for GFP and did not show positive reactivity for ALP, BSP, OPN, DSP or nestin, as shown in Figure 1B and D.

### At 21 days in the experimental group

#### Demineralized dentin tubule + DPCs + collagen gel

After 21 days, DPCs in the collagen gel assumed an osteodentin-like structure and were located along the existing dentinal wall of the demineralized dentin tubules. In the center of the tubules, morphologically, the DPCs resembled either elongated fibroblasts or odontoblast-like cells, as shown in Figure 1A.

### At 21 days in the control group

#### Demineralized dentin tubule + DPCs

At 21 days, transplanted DPCs assumed a bone-like structure and were located along the demineralized dentin tubule walls. In the center of the tubule, abundant red blood cells were observed, together with fibroblasts and capillaries, which had migrated from the surrounding rectus abdominal muscles, as shown in Figure 1C.

### Immunohistochemical observations in the experimental group at 21 days

**FIGURE 2:**
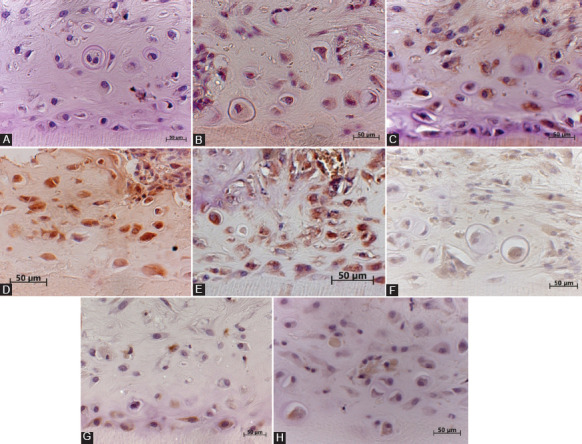
(A) The cytoplasm of odontoblast/osteoblast-like cells formed by dental pulp cells (DPCs) in the collagen type 1 scaffold inside newly formed osteodentin-like hard tissue matrix near the dentinal wall and in the center of the dentin tubule, showing positive brown staining for alkaline phosphatase (ALP) at 21 days in the experimental group; (B) the cytoplasm of osteoblast-like cells inside the bone matrix formed by DPCs without a collagen gel shows positive staining for ALP in the control group at 21 days; (C) the cytoplasm of odontoblast/osteoblast-like cells inside the osteodentin-like matrix formed by DPCs in the collagen type 1 scaffold shows positive immunoreactivity for bone sialoprotein (BSP) at 21 days in the experimental group; (D) osteoblasts inside the bone matrix formed by DPCs show positive staining for BSP in the control group at 21 days; (E) the cytoplasm of odontoblast/osteoblast-like cells inside the osteodentin-like matrix formed by DPCs in the collagen type 1 scaffold shows positive immunoreactivity for osteopontin (OPN) at 21 days in the experimental group. OPN-positive osteoblast/odontoblast-like cells are observed throughout the dentin tubule and near the dentinal wall; (F) osteoblasts inside the bone matrix formed by DPCs show weak staining for OPN in the control group at 21 days; (G) the cytoplasm of odontoblast-like cells formed by DPCs in the collagen type 1 scaffold inside newly formed osteodentin-like hard tissue matrix shows positive brown staining for nestin at 21 days in the experimental group. Nestin-positive cells are observed near the dentinal wall of the demineralized dentin tubules; (H) osteoblast/odontoblast-like cells inside the bone matrix formed by DPCs show weak staining for nestin in the control group at 21 days.

**FIGURE 3:**
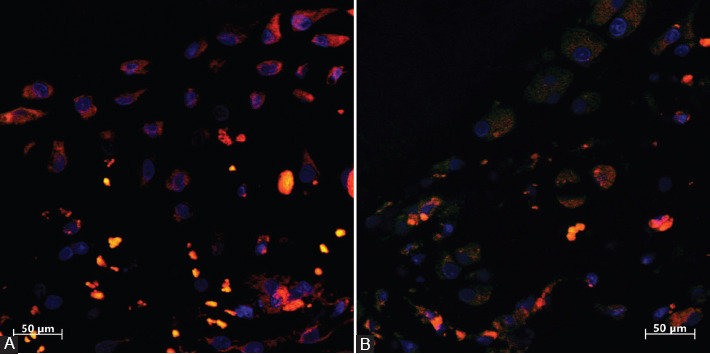
(A) The cytoplasm of odontoblast-like cells inside the newly formed osteodentin-like hard tissue matrix formed by dental pulp cells in the collagen type 1 scaffold shows positive red staining for dentin sialoprotein (DSP) at 21 days in the experimental group; (B) the cytoplasm of osteoblast-like cells inside the bone matrix shows weak red staining for DSP in the control group.

In the experimental group at 21 days, immunohistochemistry revealed that newly formed osteodentin-like, hard DPCs showed positive staining for ALP, BSP, OPN, nestin, and DSP. The cytoplasm of the DPCs was stained brown for ALP, BSP, OPN, and nestin (Figure 2A, C, E, and G) and red for DSP (Figure 3A).

### Immunohistochemical observations in the control group at 21 days

In the control group, the transplanted DPCs developed into osteoblasts, which showed weak staining for ALP, OPN, nestin and DSP, as shown in Figures 2B, F, H, and 3B. Osteoblast-like cells inside the control group showed positive brown staining for BSP, as shown in Figure 2D.

## DISCUSSION

Recent advances in translational research have improved our understanding of oral hard tissue regeneration from transplanted pulp cells [12]. In this context, the culture of DPCs appears to offer an assuring technique to warrant regeneration of the dental tissue [13]. Morphogens, scaffolds, and cells are important components of tissue engineering. However, successful regenerative strategies require rapid development of networks of functional blood vessels [1]. In this experiment, one end of each demineralized dentin tubule was left open to allow for the supply of blood and nutrition, and loaded with expanded DPCs combined with a collagen type 1 gel. The DPCs were used as responsive cells, the collagen type 1 gel as a scaffold, and the demineralized dentin tubule as a morphogen. Min et al. reported that when adult human DPCs were cultured for up to 8–9 passages and were investigated for the stemness of human adult DPCs, dentinogenic pulp cells only occurred at earlier (up to 5) passages, whereas osteogenic cells occurred throughout the whole passage range [14]. In another study, Bakopoulou and About isolated and proliferated DPSCs *in vitro* before transplantation *in vivo* for regeneration [12]. That is why we decided to use third-passage DPCs for transplantation in this study. This provided the cells with a more accessible area to secrete the extracellular matrix (ECM) and initiate the differentiation of odontoblast-like cells. Bakopoulou and About found that rat DPC populations containing stem cells exhibited the ability to differentiate into odontoblast-like cells when cultured, and they showed that this odontogenic property did not change when the cells were cultured on 3D calcium phosphate and titanium scaffolds.

In a previous *in vivo* study, it was revealed that ALP is essential for the development and potential specialization of DPCs [8]. Hard tissues, including teeth and bones, are comprised of collagen and numerous non-collagenous proteins. Several sialic acid-rich proteins, including OPN, BSP and DSP, are believed to help in the mineralization process [15]. The pattern of BSP and OPN localization is similar in hard tissues, and previous studies have indicated that ALP, BSP, and OPN are markers of mature osteoblasts/odontoblasts [8].

Recent data on GFP in transgenic mice have shown that DPSCs from incisors when transplanted into kidney capsules can lead to the formation of osteoblasts, odontoblasts, chondrocytes, and the ECM [16,17]. Ogawa et al. [18] used autogenic tooth transplantation of the coronal portion of the molar into the sublingual region in an animal model, reporting the induction of tubular dentin and bone tissue depositions. This suggests that the pulp tissue contains two important types of competent progenitor cells that differentiate either into odontoblast- or osteoblast-like cells. The presence of both lineages of osteoblasts and odontoblasts may indicate that dental pulp is comprised of numerous cell types, including resident mesoderm-derived and cranial neural crest-derived cells [19].

In the present study, at 21 days in the experimental group, an osteodentin-like hard tissue was formed, while in the control group, the DPCs formed a new bone matrix. Odontoblast-like cells were confirmed inside osteodentin-like tissue by positive immunohistochemical staining for ALP, BSP, OPN, nestin, and DSP in the experimental group. It is suggested that the scaffold can serve as the ECM for a limited period, providing a 3D framework, which allows the cells to migrate and proliferate [20]. Scaffolds can be functionalized with bioactive molecules such as growth factors to guide tissue formation or vessel network development [21]. In this study, DPCs with the collagen gel developed into osteodentin at 21 days in the experimental group, whereas that was not observed in the control group. These results are in accordance with the observations of a previous *in vitro* study, in which mRNA expression of ALP, BSP and OPN, the markers of osteogenic differentiation, increased in a 3D collagen scaffold compared to the control [8]. In the present study, the formation of an osteodentin-like matrix was observed when DPCs were transplanted with the collagen gel at 21 days in the experimental group. According to Nakashima [22], BMP-induced formation of tubular dentin is critically dependent on the scaffold. In the Nakashima study [22], odontoblast differentiation and tubular dentin formation were observed on demineralized dentin matrix inactivated by the 4M guanidine hydrochloride extraction of BMPs. Recent studies demonstrated the possibility of producing a pulp-like tissue and functional odontoblasts in several millimeters of the root canal, using stem cells from human exfoliated deciduous teeth SHED with an injectable scaffold prepared with self-assembling peptides or collagen 1 [21,23]. We used the same method in our study, which suggests that collagen type 1 gel is better in terms of accessibility inside the empty root canal compared to synthetic polymers in regenerative endodontic therapy. DPCs mixed with a collagen gel can be easily injected into an empty sterilized root canal. Dentin tubules were used as containers for the transportation of DPCs in a collagen gel in this study. According to Agha-Hosseini et al. [24], it is quite challenging to transplant human DPCs in a collagen gel into the backs of mice due to the lack of graft/implant rigidity and the manipulation of the collagen gel.

In previous studies, biologically active growth factors sequestered within dentin were released upon partial demineralization of the matrix or with the use of calcium hydroxide. This was because dentin is considered as a reservoir of “fossilized” molecules that remain bioactive and may contribute to dental pulp responses upon their release [25,26]. The results of the present study are in accordance with a previous study, in which DPCs were transplanted into mineralized dentin tubules with a collagen type 1 scaffold and were stimulated to form osteoblasts [8]. However, in the present study, an osteodentin-like matrix was observed inside the demineralized dentin tubules in the experimental groups.

The dentin matrix has also been found to contain BMP, TGF-β1, and IGF-1 activities; these cytokines are thought to be involved in the sequential differentiation of the odontoblast lineage and dentin/predentin formation in adult teeth [27]. Many studies have reported that BMPs or TGF-β specifically induce cytological and functional differentiation of odontoblast-like cells *ex vivo* and differentiation of tubular dentin-like matrix-forming cells within intrapulpal sites in *in vivo* [3,28]. BMPs have repeatedly shown a stimulatory effect in inducing osteodentin, followed by reparative dentin in mechanically exposed pulp sites [5]. Our results are in accordance with the reports mentioned above, as transplanted GFP DPCs developed into an osteodentin-like hard tissue at 21 days in the experimental groups. The transplanted GFP DPCs were confirmed by immunofluorescence staining. Huang et al. [29] reported synthesis of vascularized human pulp/dentin-like tissue in an empty human root canal space 5–6 mm deep with an open end of 2.5 mm. They observed the formation of a continuous layer of dentin-like tissue on the existing canal dentinal walls and on mineral trioxide aggregate (MTA) cement surface. In the present study, osteoblast/odontoblast-like cells were observed along the existing dentin wall in the dentin tubule, together with the formation of osteodentin after 3 weeks in the experimental group. These findings suggest that existing dentin is enough for the guidance of DPCs into odontoblasts in the dentin tubule (or empty canal). Based on our observations, it appears that DPCs embedded in the collagen matrix are attracted to the dentinal wall and differentiate into odontoblasts. The mechanism underlying this phenomenon could be the release of growth factors such as TGF-β, which would be embedded in the dentin tubule [30]. In the present study, the dentin tubules had already been demineralized with 10% EDTA before transplantation of the graft material, releasing growth factors into the dentin, especially BMP2. In light of the evidence from earlier studies, the present study indicates that transplantation of graft materials and DPCs induces differentiation of odontoblasts in the collagen gel, which then migrate toward the dentin surface and differentiate (signals from the demineralized dentin surface) into an osteodentin-like tissue.

## CONCLUSION

Our results suggest that fossilized growth factors (i.e., BPMs) are expressed from the dentin surface in the sterilized root canal. DPCs embedded in the collagen gel form an osteodentin-like hard tissue matrix in the root canal *in vivo*.
